#  The Effect of ginger herbal spray on reducing xerostomia in patients with type II diabetes

**Published:** 2017

**Authors:** Homeira Mardani, Alireza Ghannadi, Bahareh Rashnavadi, Razieh Kamali

**Affiliations:** 1 *Department of Pathology, Khorasgan (Isfahan) Branch, Islamic Azad University, Isfahan, Iran*; 2 *Department of Pharmacognosy, School of Pharmacy & Pharmaceutical Sciences, Isfahan University of Medical Sciences, Isfahan, Iran*

**Keywords:** Diabetes, Dry mouth, Ginger, Schirmer test

## Abstract

**Objective:**

The aim of the present study is to evaluate the effect of ginger herbal spray on reducing dry mouth in patients with Type II diabetes.

**Materials and Methods:**

This clinical trial was conducted on 20 patients with Type II diabetes suffering from dry mouth. The control individual for each patient was the same patient himself/herself. Each patient filled out his/her questionnaire at three different times, (before treatment, after treatment with placebo, and after taking the drug). Furthermore, the Schirmer test was performed to measure the flow of saliva in the patients. The drug and the placebo were prepared as oral sprays containing herbal extracts of ginger.

**Results:**

The mean amount of saliva after using the ginger plant spray increased significantly (p<0.001). The mean amount of saliva after treatment with medication was considerably different from the mean amount of saliva after treatment with the placebo (p<0.001). Our study included patients aged between 49 and 69 years old, (mean age 58.6 years old, and the standard deviation 5.3). The minimum and the maximum periods of type II diabetes were 2 and 21 years, the mean and the standard deviation of which were 8.8 and 5.8, respectively.

**Conclusion:**

With regard to the effectiveness of ginger herbal spray in rapidly increasing the patients’ saliva and satisfaction as well as the acceptability of this type of medicine to treat dry mouths, ginger herbal spray could act as a new, cheap, and available treatment for diabetic patients with dry mouth.

## Introduction

Diabetes, as the most common metabolic disease aside from broad systemic effects, is capable of affecting the quantity and quality of saliva and influencing oral health and dental support tissues by inducing considerable fluctuations in the metabolism of sugars and fats. Reduced volume of saliva is a regular finding in most diabetic patients that is justified by repeated urination and neurological and pathological changes in the salivary glands. The role of saliva is approved as an agent of wetting, protection, and cleaning the oral mucosa against various stimuli; therefore, any disruption in the flow of saliva, which is normally 1 mm^3^ /min, leads to difficulties in the normal activity of oral cavity (Fayaz et al., 2003 ▶).

Oral symptoms of diabetes mellitus include dry mouth, taste disturbances, parotid gland enlargement, irritation of the mucous membranes, dental caries, periodontal diseases, and bacterial and fungal infections. Studies have shown that people with diabetes have significantly worse health status, and dry mouth and periodontal diseases have higher prevalence in these individuals. Complaints about xerostomia should be separated from objective reduction of salivary flow rate. Reduced salivary flow rate follows autonomic neuropathy, especially parasympathetic. Visceral neuropathy in diabetes changes the amount of saliva by affecting the parasympathetic system and causes dry mouth( Almaskari etal., 2011 ▶).

Xerostomia, which literally means dry mouth, is defined as decrease in salivation. Xerostomia is one of the common complaints among patients. Loss of saliva function or reduction of salivation leads to dry mouth (Antonio et al., 2009 ▶). Xerostomia is often, not always, associated with decreased activity of salivary glands. Clinical findings are not always consistent with the patients’ symptoms. In some patients who complain about dry mouth, the salivary flow is sufficient to wet the mouth. In contrast, some patients with clinically dry mouth may have no complaints (Neville, 2009 ▶). Reduced salivary flow is induced by various reasons such as dehydration, stress, infection and blockage of the salivary glands, drugs, local surgery, avitaminosis, diabetes, anemia, connective tissue diseases, Sjogren’s syndrome, radiotherapy etc. (Bakhtiari et al., 2007 ▶). In anti-cancer treatment of head and neck malignancies, the role of radiation therapy is approved in the development of xerostomia. Xerostomia could be due to atrophy of the salivary glands, consumption of drugs such as antidepressants, and diseases such as diabetes (Baharvand et al., 2006 ▶). Dry mouth complications include dental problems, irritation of the mucous membranes, and impaired swallowing and speaking. Dry mouth creates many problems for the patients and severely affects the quality of life (Shirzai, 2011 ▶). In this condition, the tongue is dry, red, and atrophic, may be devoid of papilla, has atrophic gaps, and is inflamed (Bakhtiari et al., 2006 ▶). There are different methods for treatment of dry mouth, which are greatly supportive (Bakhtiari et al., 2006 ▶). The treatment is focused on wetting the mouth to stimulate saliva. For example, using oral milk, sucking small pieces of ice, and using sugar-free candies containing citric acid are non-medicinal treatments, and the use of pilocarpine as a pharmaceutical method can alleviate this condition. Saliva substitutes or artificial saliva is also employed to keep the mouth wet, but has not been accepted by a large number of patients and is expensive (Fayaz et al., 2003 ▶). To relieve symptoms and discomfort of dry mouth, it is possible to sip water throughout the day. The use of caffeine should be limited, and alcohol-containing mouthwash should be discontinued. In addition, the use of Vaseline on the lips is appropriate. The general treatment of dry mouth is the salivary substitutes or stimulants, and since such saliva substitutes do not exist in Iran, mouthwash is recommended. It is also possible to use oral balance solutions available in the market (Bakhtiari et al., 2007 ▶). The use of medical herbs to treat diseases dates back to the first years of human life, and this type of treatment has been evolved and developed over the centuries. Several thousand years of history of medicine and pharmacy records indicate valuable experiences and information in the field of herbal therapy (Amin, 1991 ▶). The interesting point is that spices do not have nutritional value or are incomplete; however, ginger has healing properties that have been studied in today’s nutritional science (Rezai, 2012 ▶). 

Since 2000 BC, ginger has been used in traditional Chinese and Indian medicine. Ginger is beneficial in digestion and rheumatic inflammation (Minaiyan et al., 2008 ▶) and has been widely utilized in Chinese herbal medicines around the world. Studies have approved the following effects of ginger: soothing and anti-inflammatory effects in rheumatoid arthritis (Bahmani et al., 2013 ▶; Nakhaei et al., 2012 ▶; Minaiyan et al., 2008 ▶; Altman et al.,2001 ▶), the inhibitory effect on inflammatory mediators and pathways (Bahmani et al., 2013 ▶; Lantz et al., 2007 ▶; Zhou et al., 2006 ▶), analgesic effect (Bahmani et al., 2013 ▶; Terry et al., 2011 ▶), and anti-nausea and anti-vomiting effect (Minaiyan et al., 2008 ▶; Bahmani et al., 2013 ▶; Parsa et al., 2011 ▶; Sontake et al., 2003 ▶). Of course, more studies are required to approve the use of ginger as an anti-nausea drug during chemotherapy (Handiadka et al., 2012 ▶).

Ginger is also employed in the treatment of dental pain (Bahmani et al., 2013 ▶; Sudarshan et al., 2012 ▶), and for stimulating the salivation, oral thrush treatment (*Candida albicans*), oral anti-cancer treatment, herpes simplex infection treatment (Sudarshan et al., 2012 ▶), and antimicrobial activity against teeth cariogenic bacteria (Patal et al., 2011 ▶).

Ginger has been shown as a harmless herbal medicine, the side effects of which are rare and insignificant (Mallikarjuna et al., 2008 ▶). Concerning ginger, its compounds, and its harmful effects within a long period of time, more analyses are required to be done on animals and humans (Nakhaei et al., 2012 ▶). Ginger volatile oil is reported to be free from irritating and allergenic effects, but there is a risk of dermatitis in sensitive individuals. Large quantities of ginger may interact with drugs used by heart-disease patients and anti-clotting drugs. During pregnancy and lactation, it is better to avoid taking the amounts more than the ordinary diet (Minaiyan et al., 2008 ▶). 

Diabetes mellitus is the most common metabolic disease. Its prevalence is increasing in the world, and based on the studies, the rate of growth in Type II diabetes might be higher than that of Type I diabetes. Researchers have been trying to take advantage of the properties of medicinal plants in modern medicine. Furthermore, people in developed countries have abandoned synthetic chemical drugs and have turned to herbal remedies. Therefore, a thorough investigation on the proper application of these plants, especially in circumstances where the application of medicinal plants in pharmaceuticals, cosmetics, health, and food is accelerated, is essential. While traditional medicine has not moved forward as fast as modern medicine, developments in the field of traditional medicine and medicinal plants. Due to the abundance and availability of plant species in Iran, obtaining a preparation that can stimulate the salivation is necessary. The present study examines the effect of ginger plant spray on reducing dry mouth in patients with type II diabetes. In this study, the drug and the placebo were provided in the form of spray to control the daily intake of patients and to make their application easier to be used anywhere and at any time. Also, taking the medication led to greater cooperation of the patients, and the good feeling of fresh mouth after spray encouraged them to apply the drug and the placebo. Moreover, since the patients were used to wash their mouth after using the mouthwash, spray preparation of the drug and the placebo solved this problem. Thus, in this study, we address the effect of ginger herbal spray on dry mouth in people with Type II diabetes.

## Materials and Methods

Twenty patients with type II diabetes suffering from dry mouth were selected from Zarrin Diabetes Center, Isfahan, Iran. Forty samples were studied in two groups. To reduce the disruptive factors, the control group included the same subjects. The patients who suffered from various degrees of dry mouth were selected, using a questionnaire and the Schirmer test. Medical records of the patients were studied. The inclusion criteria were as follows: patients with type II diabetes with HbA1c ≤ 73 with dry mouth and Schirmer test ≤ 25 mm. The exclusion criteria were as follows: allergy to ginger, pregnancy, the use of anticoagulant drugs such as warfarin or aspirin, smoking, drug abuse, using antidepressants, other systemic diseases, HIV positive and connective tissue diseases such as Sjogren’s, history of radiotherapy and chemotherapy, the patients who had used saliva-enhancing drugs before the treatment, and the patients who refused to fill out the consent form. The patients’ information, including personal information such as name, surname, age, sex, duration of diabetes, type of treatment, fasting blood glucose, drugs being used and other conditions like thyroid diseases, blood lipids profile, blood pressure, and disorders of heart, kidney, and eye were recorded based on the answers, and the study of medical records of the patients. All subjects had profiles in the diabetes center, and the history of their disease was recorded. The patients’ tongue, mouth, lips, and cheeks were examined. 

For a more detailed assessment, the patients were classified into 4 groups, of 5 subjects. The values measured after the initial Schirmer test for groups A, B, C, and D were 0-6, 7-12, 13-19, and 20-25, respectively. 

The drug was prepared as an oral spray and the drug solution contained 1/3 of ginger extract (Bakhtiari et al., 2007 ▶), 1/3 edible glycerin, and 1/3 distilled water. 

The placebo (Bakhtiari et al., 2007 ▶) was prepared as an oral spray that contained 1/3 edible glycerin, and 2/3 distilled water.

**Ginger extracts preparation**

Ginger root (*Zingiber officinale*) was a gift from Gol Daru Pharmaceutical Company (Isfahan, Iran) received in September, 2013, and approved by Pharmacognosy Department of Isfahan School of Pharmacy. Then, 300 g of ginger root was powdered and mixed with 1500 cc of 70% ethyl alcohol for 3 days. After this period, the mixture (powder and 70% alcohol) was placed in a shaker for 2 hours. Following that, the filtration process was conducted, the alcohol in the final extract was removed by rotary evaporator, and the ginger-concentrated extract was obtained. The extraction method in this study was the same as that of Hajhashemi et al. (Hajhashemi et al., 2003 ▶). The concentrated extract of ginger was refrigerated until the preparation of the spray.

**The Schirmer test**

The Schirmer test is a simple method to measure the production of tears. The Schirmer strips are disposable paper strips with standard size of 35 mm (Riordan et al., 2010 ▶) and are used by ophthalmologists to measure the moisture of the eyes. They are commercially available in sizes of 35×5mm. The developed routine Schirmer test has a blue bar that moves with the fluid and determines the fluid flow rate (Khovidhunkit et al., 2009 ▶; Dyasanoor et al., 2014 ▶).

**Data completion by the Schirmer test**

The completion method using a questionnaire and the Schirmer test was performed in 3 steps. Completing the questionnaire and conducting the Schirmer test were done in the first step and after using the drug and the placebo. Subsequently, the placebo plus explanations and written clarifications (written on the spray) on how to use it were given to the patients. The patient were given a second appointment 10 days after using the placebo. Five days after using the spray, we contacted the patients and the correct application of the spray was ensured. Filling out the questionnaire and the Schirmer test were done in the second step 10 days after taking the placebo. Afterwards, the drug plus explanations and written clarifications (written on the spray) on how to use it were given to the patients. Similar to the placebo step, 5 days after using the spray, we contacted the patients and the correct application of the spray was ensured. Filling out the questionnaire and the Schirmer test were done in the third step 10 days after taking the placebo. During the study, patients did not take herbal and chemical medicine affecting the amount of saliva, and did not use mouthwashes, or artificial saliva.

**How to complete the questionnaire?**

The questions of the questionnaires (a) and (b) were designed on the basis of the questions in Chavez et al. (Terry et al., 2011 ▶), Rabie et al. (Khovidhunkit et al., 2009 ▶), and Rad et al. (Burket, 2010 ▶) inventories. We prepared two questionnaires. The questions of the questionnaire (a) (selected for the patients with dry mouth) and the questionnaire (b) (selected for the severity of dry mouth) were asked to obtain full information of the patients.

**How to measure the amount of saliva by the Schirmer test?**

In order to unify the conditions in all phases of the study, saliva measurement was conducted between the hours 10-12 a.m. The patients were present 2 hours before taking the test and were prohibited from eating, drinking, or smoking. In order to measure unstimulated salivary flow, sterile Paper bar of the Schirmer test was applied. The patients were asked to swallow all saliva before taking the test, but not to swallow their saliva during testing. In addition, the patients were asked to stick their tongue to their palate and place the tip of the tongue behind their upper teeth so that the test paper would not touch the tongue while measuring the saliva. The round end of the bar was on the floor of the mouth, and the test was kept by forceps vertically for 3 min. When the rounded end of the bar touched the floor of the mouth moisture, it was moved to the top of the bar. After 3 min, the test was removed from the mouth, and the wet length was read and recorded quickly. If the wet length was ≤ 25, the patient had dry mouth; otherwise, the patients was excluded from the study and replaced with another sample. 

**Statistical analysis**

Repeated measure ANOVA was applied in order to compare the change in dry mouth at three times, including before the treatment, after the treatment with placebo, and after taking the drug. Additionally, the LSD test was applied to compare the mean change between the times.

## Results

The number of the subject was 20, (7 male (35%) and female13 (65%)). Our study included patients aged between 49 and 69, the mean of which was 58.6 years old, and the standard deviation was 5.3. The minimum and the maximum periods of type II diabetes were 2 and 21 years, the mean and the standard deviation of which were 8.8 and 5.8, respectively. 

**Table 1:** Average standard deviation of minimum and maximum range

**Variable **	**Mean±SD**
**Age **	58.6±5.3
**Diabetes duration**	8.8±5.8

Data are shown as Mean±SD

**Table 2:** Distribution of treatment for diabetic patients

**p 2**	**p 1**	**Mean±SD**	**Time **
		8.3±2.2	**Before the treatment **
	0.163	7.9±2.5	**After taking the placebo **
0.0006	0.0000	2.4±1.2	**After taking the drug **

Data are shown as No. (%)

Sixteen patients (80%) were using tablets, and 4 patients (20%) were taking tablets and insulin to treat their diabetes. Repeated measure ANOVA indicated that the mean Schirmer test at three times, including before the treatment, after the treatment with placebo, and after drug use was not the same (p<0.001). Meanwhile, LSD *post hoc* test showed that the mean Schirmer tests before the treatment and after the treatment with placebo were not significantly different (p1=0.12). However, the mean Schirmer test using the drug significantly increased compared to the placebo (p<0.001). (Table 3).

**Table 3:** Average Schirmer test of patients at different times

**Time **	**Mean±SD**	**p 1**	**p 2**
**Before the treatment **	8.3±2.2		
**After taking the placebo **	7.9±2.5	0.163	
**After taking the drug **	2.4±1.2	0.0000	0.0006

P1 is the p value of comparing after the treatment with before treatment.

P2 is the p value of comparing after taking the placebo with after taking the drug.

Repeated measure ANOVA demonstrated that the mean score of dry mouth at the three times was not the same (p<0.001). Meanwhile, the LSD *post hoc* test indicated that the mean dry mouth before and after the treatment with placebo had no significant difference (p=0.163). However, the mean score of the selected patients with dry mouth after the treatment with the drug was significantly reduced compared to the placebo (p<0.001) (Table 4)

**Table 4:** Average score of dry mouth in patients

**Diabetes treatment method**	No (%)
**Tablet**	16(80)
**Pills and insulin**	4(20)
**Total**	20(100)

P1 is the p value of comparing after treatment with before treatment.

P2 is the p value of comparing after taking the placebo with after taking the drug.

**Table 5:** Severity of dry mouth in patients at different times

**Time **	**Mean±SD**	**p 1**	**p 2**
**Before the treatment **	19.1±6.3		
**After taking the placebo **	17±5.7	0.11	
**After taking the drug **	5.2±3.02	0.0002	0.0000

P1 is the p value of comparing after treatment with before treatment.

P2 is the p value of comparing after taking the placebo with after taking the drug.

Repeated measure ANOVA revealed that the mean score of dry mouth severity at three times was not the same (p<0.001). Meanwhile, the LSD *post hoc* test showed that the mean severity of dry mouth before and after the treatment with the placebo had no significant difference (p=0.11). However, the mean score of severity of dry mouth after the treatment with the drug was significantly reduced compared to the placebo (p<0.001).

Spearman correlation coefficient showed that there is a significant relationship between the amounts of classes for the Schirmer test (A-D) and the changes in severity of dry mouth (p<0.001, r=0.704).

As shown in Table (6), the reduction in the severity of dry mouth was more significant at lower classes. This means that the severity of dry mouth reduction decreases from A to D.

**Table 6:** Mean change of dry mouth after using drugs classified according to the Schirmer tes

	**The Schirmer test Classification**
	**Mean±SD**
**A**	-15.6±3.4
**B**	-11.8±4.7
**C**	-11±1.6
**D**	-8.6±2.9

T -test showed that the mean Schirmer test changes (P=0.39) and dry mouth (P= 0.41) did not differ significantly between male and female patients. Pearson correlation coefficient illustrated that there was a significant relationship between FBS (P=0.006), HbA1C (P=0.03), disease duration (P=0.04), age (P=0.04), and changes in dry mouth severity (efficiency), as this correlation coefficient had lower efficiency among people with higher FBS and hba1c and older age. 

In the present study, we addressed the effect of ginger herbal spray on dry mouth in patients with type II diabetes. The results of our study showed that the mean Schirmer test at three times (before the treatment, after the treatment with the placebo, and after using the drug) was not the same. Meanwhile, the LSD *post-hoc* test indicated that the mean Schirmer test before and after taking the placebo was not significantly different. However, the average Schirmer test after using the drug, significantly increased compared to the placebo. These data suggest that the value of the Schirmer test was increased after taking the ginger herbal spray, representing an increase in salivating after using the ginger spray.

**Table 7:** Mean change in Schirmer test among patients with dry mouth after using the drug based on gender

	** Male **	**Female**	**P**
	Mean±SD	Mean±SD	
**The Schirmer test**	7.6±3.4	9.1±3.9	0.39
**Change of dry mouth score**	-6.1±1.2	-5.2±2.6	0.44
**Change of severity of dry mouth score**	-10.7±4.2	-12.3±4	0.41

## Discussion

The above-mentioned data reveal that a sense of the severity of dry mouth was releived in patients after taking the ginger herbal spray. These data suggest that ginger is influential in increasing salivation and reducing xerostomia in patients. Alaei et al. (2009) ▶ demonstrated that ginger is effective in improving dry mouth in patients after head and neck irradiation. They gave their patients ginger tablets 3 times a day for 2 weeks. they analyzed the systemic effects of ginger on dry mouth in patients with head and neck radiation. The results of the present study are consistent with those of the study conducted by Alaei et al.; however, in their recent study they used tablets, while we used it in the form of spray. Since the patients with dry mouth were old and took multiple medications to treat their disease. Fayyad and Attar-Zadeh (2003) indicated that the product obtained from the seeds of basil can be used in patients with dry mouth as a replacement for saliva. In our study, however, the materials used had local effects only, and none of them contributed to salivation. 
